# Age matters: Microbiome depletion prior to repeat mild traumatic brain injury differentially alters microbial composition and function in adolescent and adult rats

**DOI:** 10.1371/journal.pone.0278259

**Published:** 2022-11-30

**Authors:** Marissa Sgro, Giulia Iacono, Glenn R. Yamakawa, Zoe N. Kodila, Benjamin J. Marsland, Richelle Mychasiuk

**Affiliations:** 1 Department of Neuroscience, Central Clinical School, Monash University, Melbourne, Victoria, Australia; 2 Department of Immunology and Pathology, Central Clinical School, Monash University, Melbourne, Victoria, Australia; UAE University: United Arab Emirates University, UNITED ARAB EMIRATES

## Abstract

Dysregulation of the gut microbiome has been shown to perpetuate neuroinflammation, alter intestinal permeability, and modify repetitive mild traumatic brain injury (RmTBI)-induced deficits. However, there have been no investigations regarding the comparative effects that the microbiome may have on RmTBI in adolescents and adults. Therefore, we examined the influence of microbiome depletion prior to RmTBI on microbial composition and metabolome, in adolescent and adult Sprague Dawley rats. Rats were randomly assigned to standard or antibiotic drinking water for 14 days, and to subsequent sham or RmTBIs. The gut microbiome composition and metabolome were analysed at baseline, 1 day after the first mTBI, and at euthanasia (11 days following the third mTBI). At euthanasia, intestinal samples were also collected to quantify tight junction protein (*TJP1* and *occludin*) expression. Adolescents were significantly more susceptible to microbiome depletion via antibiotic administration which increased pro-inflammatory composition and metabolites. Furthermore, RmTBI induced a transient increase in ‘beneficial bacteria’ (*Lachnospiraceae* and *Faecalibaculum*) in only adolescents that may indicate compensatory action in response to the injury. Finally, microbiome depletion prior to RmTBI generated a microbiome composition and metabolome that exemplified a potentially chronic pathogenic and inflammatory state as demonstrated by increased *Clostridium innocuum* and *Erysipelatoclostridium* and reductions in *Bacteroides and Clostridium Sensu Stricto*. Results highlight that adolescents are more vulnerable to RmTBI compared to adults and dysbiosis prior to injury may exacerbate secondary inflammatory cascades.

## Introduction

Repetitive mild traumatic brain injury (RmTBI) accounts for a large proportion of traumatic brain injuries (TBI) worldwide and subsequently results in large medical and economic burdens to the healthcare system [1]. Individuals that experience RmTBI are at increased risk for cognitive impairments, mental health disorders (e.g. depression and anxiety) [2], and gastrointestinal dysfunction [3–5]. The probable mechanisms leading to these deficits include secondary inflammatory cascades whereby persistent pro-inflammatory states occur systemically and within the brain, resulting in prolonged microglia activation and alterations to hypothalamic-pituitary-adrenal (HPA) axis functioning [2]. Importantly, adolescents are at the highest risk for sustaining RmTBI [6,7], with previous research demonstrating that 1 in 5 youth will have sustained a mild TBI (mTBI) by 16 years of age. Given that this time period is critical for brain development, adolescents exhibit more post-RmTBI deficits and require longer periods of time to recover [8,9]. The heterogeneity identified in symptomologies following adolescent RmTBI necessitates further investigations into the pathophysiological mechanisms involved in the development of these deficits. However, to date, studies have primarily focused on adult populations, specifically when examining systemic influences on mTBI outcomes.

Interestingly, recent literature has demonstrated that mTBI influences the gut microbiome and the brain-gut axis, whereby alterations and injury-induced dysbiosis to the gut microbiota further perpetuate neuroinflammation, alter intestinal permeability, and modify mTBI-induced deficits [10]. Under steady-state conditions, gut bacteria are important in the maintenance of intestinal homeostasis and immune system functioning, via the regulation of inflammatory pathways [11]. However, studies have shown that mTBI-induced dysbiosis may exacerbate deficits in gut microbiome functionality by modulating the intermediary mediators of the brain and gut; known as metabolites [12]. Rodent studies investigating the impact of RmTBI on the gut microbiome have also identified alterations to vagal nerve function [13], HPA axis [14], short chain fatty acid (SCFA) production, microglia activation [15], and intestinal and blood brain barrier (BBB) permeability [16], post injury.

Although studies in adult rodents have demonstrated both transient [17] and chronic [18] changes in gut microbiome composition and functionality following RmTBI, there have been no such studies in adolescents. Furthermore, there has been a complete lack of investigation into the effects of microbiome dysfunction prior to RmTBI, in either adolescents or adults. Therefore, we sought to examine how microbiome depletion prior to RmTBI influences the composition and functionality of the gut microbiome in adolescent and adult Sprague Dawley rats. To do so, we induced microbiome depletion by administration of a broad-spectrum antibiotic cocktail for 14 days prior to RmTBI induction in adolescent and adult rats. Gut microbiome composition via 16S rRNA gene amplicon sequencing and functionality (metabolomics) via untargeted LC-MS technology was analysed at baseline, 1-day post-injury administration (day 17), and 11-days post injury (day 30 or euthanasia). Furthermore, tight junction protein expression of intestinal samples were analysed to identify changes in intestinal permeability and/or mucosal structural integrity.

## Methods

### Animals and gut microbiome depletion treatment

**Fig 1:**
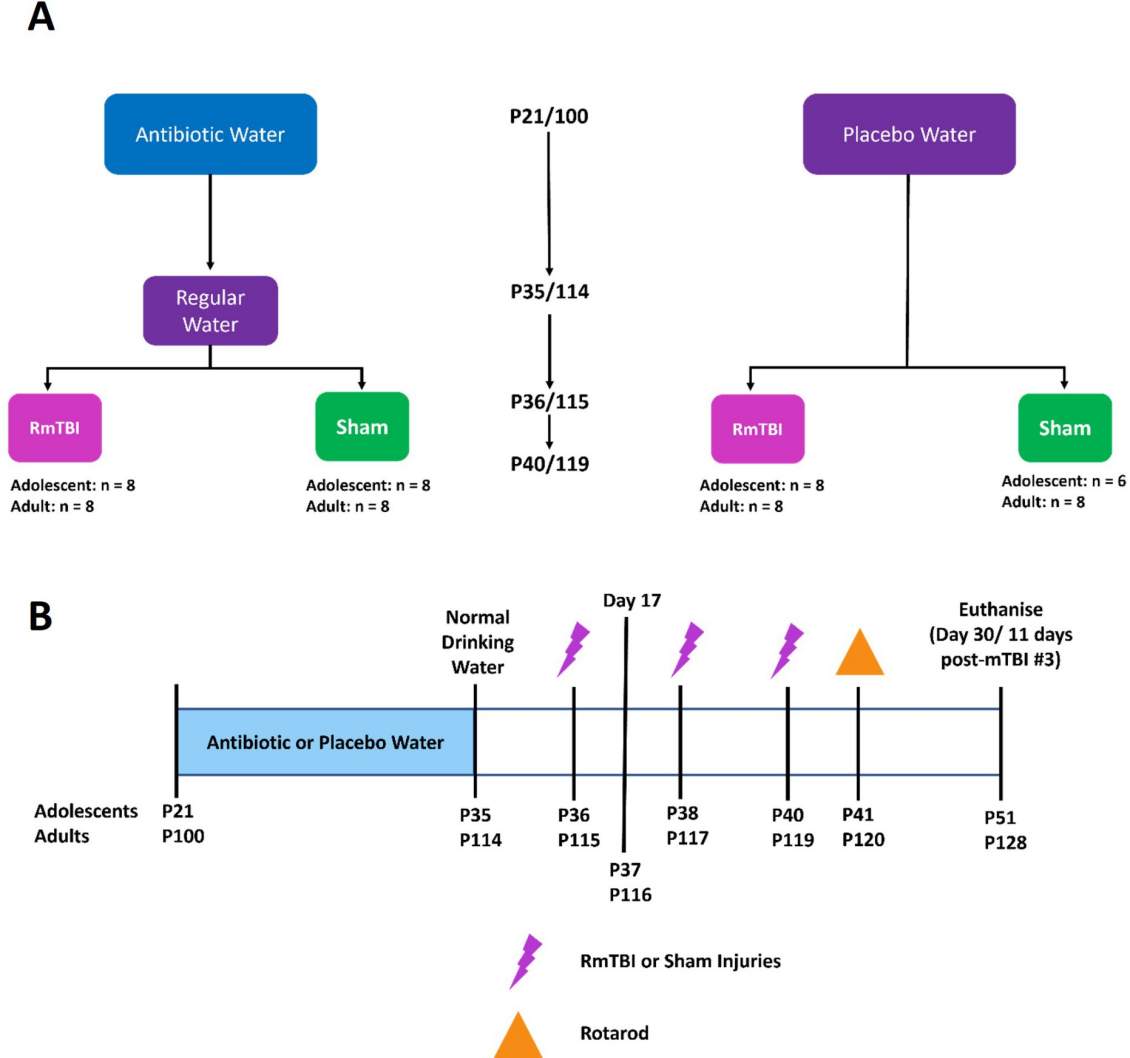
A) Flowchart detailing experimental groups and group sizes; B) Experimental timeline illustrating order and day of procedures. Fecal samples were collected at baseline, day 17, and day 30 were utilized for 16s rRNA sequencing and metabolomics.

All animal procedures were approved by the Alfred Medical Research and Education Precinct Animal Ethics Committee and carried out in accordance with the Precinct Animal Centre (E/1992/2020/M). Male and female Sprague Dawley adolescent and adult rats were acquired from the Monash Animal Research Platform. All animals were maintained on a 12:12 hr light:dark cycle, with lights on at 0600, in a temperature controlled (21°C) facility. At postnatal (P) day 21 and 100, adolescent (n = 31) and adult (n = 32) Sprague Dawley rats, were randomly assigned to either antibiotic (17 adolescents, 16 adults) or standard autoclaved drinking water (14 adolescents, 16 adults); equal males and females were included in each group. The antibiotic cocktail administered over the 14 days in their drinking water consisted of ampicillin (1g/L), vancomycin (500mg/L), imipenem (250mg/L), metronidazole (1g/L), and ciprofloxacin HCL (20mg/L). This antibiotic cocktail was previously described in Hoban et al,^23^ to effectively deplete the gut microbiota. Rats on antibiotic water were transferred back to standard autoclaved drinking water on P36/115 (adolescent/adult) for the remainder of the study. See Fig 1 for group sizes and experimental timeline. Group sizes were determined with sufficient *a priori* statistical power using effect sizes generated from similar studies within our laboratory.

### RmTBI induction–lateral impact device

**Fig 2:**
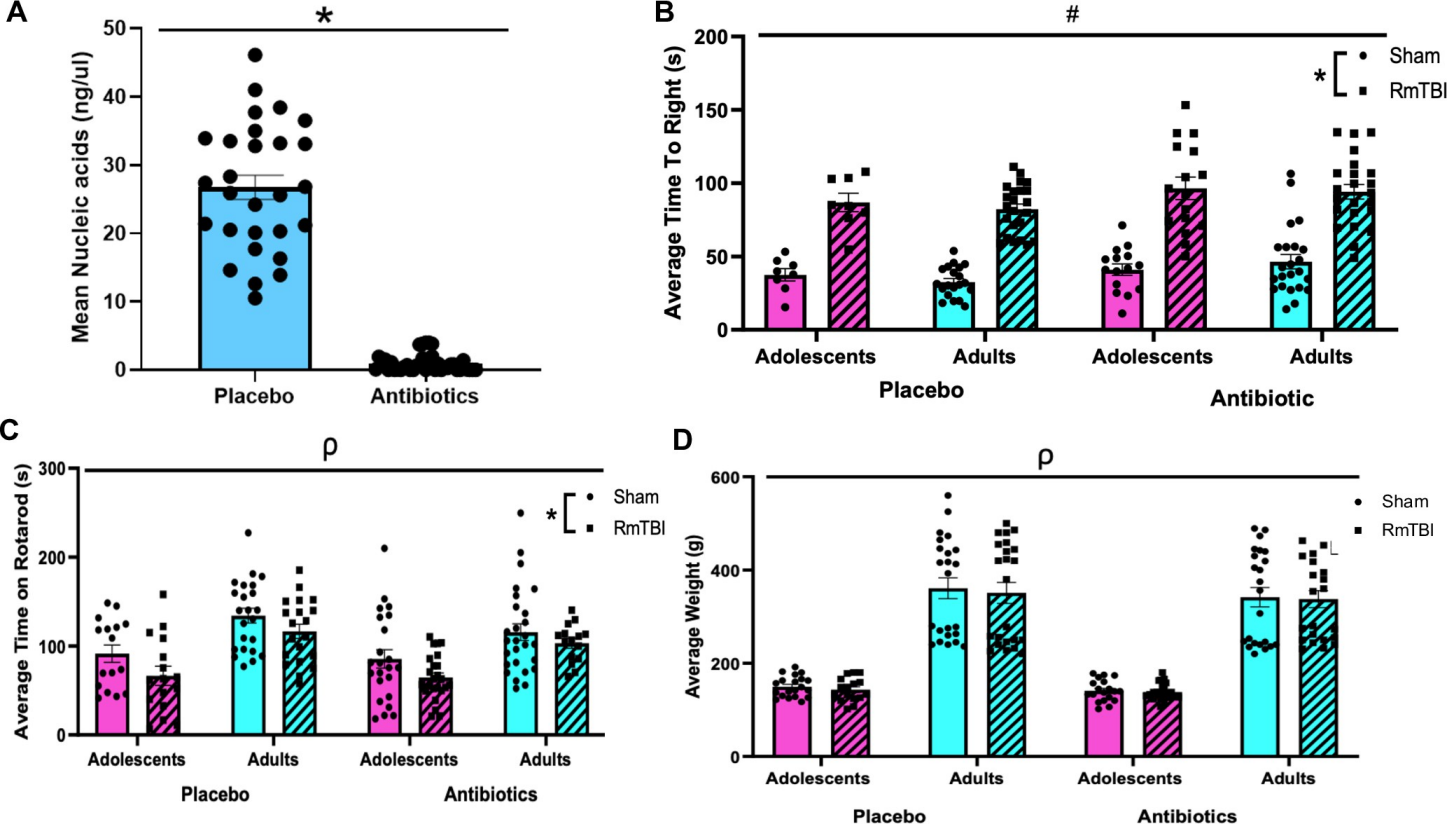
Animal characteristics. (**A)** Confirms depletion of the gut microbiome, as rats on the antibiotic cocktail had a significantly reduced mean nucleic acid concentrations **(B,C)** Confirm injury induction as rats that received RmTBI demonstrated significantly longer time-to-rights compared to shams **(B)** and rats that received RmTBI had significantly shorter times on rotarod compared to shams **(C).** Finally, adult rats weighed more than adolescents, but antibiotic treatment and RmTBI did not affect average weight **(D)**. Graphs are shown as means ± SEM, * represent significant difference, *p* < .05.

Rats in each group were randomised to receive 3 mTBIs (17 adolescents, 16 adults) or sham injuries (14 adolescents, 16 adults) at P36/P115, P38/P117, and P40/P119; adolescents/adults respectively. Adolescents: (Placebo + Sham; n = 6), (Placebo + RmTBI; n = 8), (Antibiotic + Sham; n = 8) (Antibiotic + RmTBI; n = 9), and Adults: (Placebo + Sham; n = 8), (Placebo + RmTBI; n = 8), (Antibiotic + Sham; n = 8), (Antibiotic + RmTBI; n = 8). Sham and RmTBI injuries were administered using the lateral impact (LI) device, as previously described [19]. Specifically, rats were anaesthetized with 5% isoflurane and placed chest down on a low friction Teflon® board. With the left temporal lobe facing the impactor, a 50-gram weight was propelled toward the rat’s head using pneumatic pressure at an average speed of 8.39 ±0.31 m/s and 8.926 ± .15 m/s, inducing mTBIs at ~85.52 Gs for adolescents and ~90.09 Gs for adults. The weight impacted a small aluminium ‘helmet’ to prevent damage to the skull whilst propelling the rat into a horizontal 180° rotation across the Teflon® board. Following impact, the rat was removed from the device and placed on its back in a clean, warm cage to recover. This LI technique is a clinically relevant model that emulates the acceleration and rotational forces of sports-related concussion [19]. The amount of time required for the rat to wake and flip from a supine to a prone position (time-to-right) was recorded as a measure of loss of consciousness. See Fig 2.

### Confirmation of gut microbiome depletion

Gut microbial DNA concentrations were obtained by collecting stool samples from the commencement of antibiotic administration to the completion of the study. After collection, stool samples were stored at -80°C. Samples were thawed, and gut bacteria were extracted using QIAGEN QIAamp Fast DNA Stool Mini Kit as described by the manufacturer’s instructions (Qiagen, Hilden Germany). The concentration and quality of bacterial DNA was measured with the QIAGEN QIAxpert Spectrophotometer using an absorbance ratio of A260/280 (Qiagen, Hilden Germany). See Fig 2.

### Confirmation of injury induction

The rotarod is a widely utilised and standardised method of assessing motor function and was used to confirm injury induction. The apparatus consisted of a rotating barrel divided by walls into four equal lanes. Rotarod testing was completed by a researcher blinded to all experimental conditions. Rats were pre-trained to remain on the Rota-Rod for 2 min. Each rodent was given 3 training sessions at a constant speed of 4 rpm for 2 minutes. The third trial session is 2 minutes on the accelerating mode. Here the machine slowly accelerated up to ~40 rpm over a 5 min period. The speed at 2 minutes is ~15 rpm. If a rodent fell off the Rota-Rod the timer was stopped. There were 3 testing sessions on the accelerating Rota-Rod at 4 rpm. Over the 5 minutes, the Rota-Rod built up to a speed of 40 rpm. When the 5 minutes was over any animals remaining on the Rota-Rod were returned to their home cage and awaited testing sessions 2 and 3. The 3 trials were then averaged to generate an average time on the Rota Rod out of 300 seconds. See Fig 2.

### Microbiome 16S rRNA gene amplicon sequencing

#### Microbial DNA extraction and library preparation

Bacterial DNA was extracted from rat pellets using the QIAamp PowerFecal Pro DNA Kit (Qiagen, 51804) according to the manufacturer’s protocol and eluted in 40μl microbial DNA-free water. Microbial environmental contamination was controlled using negative extraction controls using DNA kit extraction buffers only. Extraction efficiency was controlled using 5μl ZymoBIOMICS Microbial Community Standard (D6300, Integrated Sciences). DNA extraction was performed under a PCR hood using sterile and microbial DNA/DNAse-free material. Extracted microbial DNA was amplified using custom barcoded primers targeting the V1-V2 hypervariable region of the bacterial 16S rRNA gene (F-27/R-338) as previously described [20]. Primers were as following, with N sequences as sample-specific 12-nucleotides golean barcodes:

16S-Forward: 5’AATGATACGGCGACCACCGAGATCTACACTATGGTAATTCCAGMGTTYGATYMTGGCTCAG-3’;
16S-Reverse: 5’CAAGCAGAAGACGGCATACGAGATACGAGACTGATTNNNNNNNNNNNNAAGCTGCCTCCCGTAGGAGT-3’;

Each 25μl PCR reaction was composed of 2μl of DNA (or negative or positive extraction control), 18.4μl of microbial DNA-free water, 1μl of forward and reverse primers at 5μM, 2.5μl of Accuprime PCR buffer II, 0.1μl Accuprime TAQ High Fidelity DNA polymerase (12346086, Life Technologies). Each run included 2 negative reaction controls using microbial DNA-free water (338132, Qiagen). PCR was performed under a PCR hood using sterile and microbial DNA/DNAse-free material by a researcher blinded to experimental conditions. Cycling parameters: initial denaturation 3 min at 94°C, followed by 30 cycles of 30 s denaturation at 94°C, 30 s annealing at 56°C and 60 s elongation at 68°C, with a final extension at 68°C for 10 min. A High Sensitivity NGS Fragment Analysis Kit (ATI-DNF-474-0500, Integrated Sciences) with a 12-capillary Fragment Analyser System (5200, Agilent) was used to quantify amplified PCR amplicons. PCR amplicons were pooled at equimolar concentrations of 3nM and purified using Agencourt AMPure XP beads (A63881, Beckman Coulter). Denatured amplicon libraries were sequenced using the MiSeq Illumina technology (MiSeq Reagent Kit v2–500-cycles, 2x 250bp, 20% PhiX).

#### Data analysis

Raw sequencing data was processed using the dada2 pipeline from the dada2 package (version 1.16.0) [21]. Forward and reverse reads were truncated using *truncLen = c(240*, *240)*, *maxEE = c(2*,*5) and trunQ = c(2*,*2)*. The remaining parameters were set as default. Bacterial taxonomy was assigned against the SILVA 16S rRNA database (version 138) [22]. Sequences were aligned using the *DECIPHER* package (version 2.18.1) [23]. The *phangorn* package (version 2.5.5) [24] was then used to construct a maximum likelihood phylogenetic tree. A read threshold of 300 reads per sample was then applied to allow the inclusion of low-read antibiotic-treated samples and exclude negative extraction and PCR controls. Five antibiotic-treated samples were below this threshold and had to be excluded. The final dataset included 1921 ASVs and 177 samples; ASVs were distributed among 9 phyla with *Firmicutes*, *Bacteroidota* and *Actinobacteroidota* as the most abundant; and 111 genera, including *Bacteroides*, *Lachnospiraceae*, *Turicibacter*, *Alistipes* and *Faecalibaculum* as the most abundant. The Shannon alpha-diversity index was calculated using the *phyloseq* package (version 1.34.0) [25]. Microbial datasets were cumulative sum scaled normalised and log transformed (logCSS) using the *metagenomeSeq* package (version 1.32.0) [26]. Weighted Unifrac dissimilarities were calculated using the *phyloseq* package (version 1.34.0). Investigation of factors explaining microbial variation was performed using the permutational multivariate analysis of variance (PERMANOVA) method using the *adonis* function of the *vegan* package (version 2.5–7) on weighted Unifrac dissimilarities (999 permutations) [27]. The *Limma* package (version 3.50.1) [28] and the *MaAsLin2* package (version 1.6.0) [29] were used to detect differentially expressed ASVs. For visualisation purposes, the dataset was filtered using a detection threshold of 10 reads and 0.05 prevalence when visualising taxonomic composition at the genus level.

### Metabolomics mass spectrometry

#### Extraction and acquisition

600 μl cold extraction solvent (containing 1μM CCTP internal std, 5μM BHT, 1/1000 15N, 13C-Amino Acid mixture in Methanol (1060351000, Sigma-Aldrich)) was added to 20 mg of crushed stool pellets and samples were mixed at 4C for 1h. Blanks made with water (338132, Qiagen) were extracted alongside. Samples were centrifuged at 20’000g for 10 min. Supernatants were transferred to LC-MS vials. Supernatant leftovers were combined to make a pooled QC sample. Samples were randomised and acquired at the Monash Proteomics and Metabolomics Facility in Parkville, Melbourne Victoria Australia. A QExactive Orbitrap mass spectrometer (Thermo Scientific) coupled with the Dionex Ultimate® 3000 RS (Thermo Fisher) high-performance liquid chromatography (HPLC) system was used to acquire LCMS data. Chromatographic separation was performed on a ZIC-pHILIC column (5μm, polymeric, 150 x 4.6mm, SeQuant®, Merck).

#### Data analysis

Raw Thermo Fisher sample files were deconvoluted, aligned, and annotated using MS-DIAL (version 4.7) following a custom pipeline https://github.com/respiratory-immunology-lab/metabolome-lipidome-MSDIAL [30]. Data was annotated against the MassBank negative and positive databases (Accessed in November 2021) [31]. Parameters used were as follows: MS tolerance: 0.003; Min peak height: 100’000; Rt tolerance: 2min; Gap filling: TRUE. Total metabolites identified: positive mode, 42377; negative mode, 16112. The pmp package (version 1.4.0) [32] was used to filter samples by blanks (*fold_change = 2*, *fraction_in_blank = 0*.*77*) and for feature prevalence across samples (*min_frac = 0*.*38*, *method = ‘across’*), and within (quality control) QC samples with the filter_peaks_by_rsd (*max_rsd = 60*). QCRSC was used to correct for signal drift (*spar = 1*, *minQC = 4*). The filtered dataset contained 1034 metabolites. Following probabilistic quotient normalisation, missing values were imputed using the *“knn”* method of imputation, and data was scaled using the generalised logarithm method. Metabolites were annotated using the GNPS, HMDB and MS-DIAL databases. All metabolites were manually screened for raw spectra quality within MS-DIAL, and 182 reliable annotated metabolites were retained for downstream analysis. Investigation of factors explaining metabolomic variation was performed using the permutational multivariate analysis of variance (PERMANOVA) method using the *adonis* function of the *vegan* package (version 2.5–7) on Euclidean distances (999 permutations). The *Limma* R package (version 3.50.1) was used to detect differentially expressed metabolites.

All analyses were performed using R (version 4.1.3) [33] by an experimenter blinded to all research conditions and manipulations. A *p* < 0.05 and an adj. *p* < 0.05 were considered statistically significant. P-values were corrected for multiple testing using the Benjamini-Hochberg (BH) method. Plots were plotted using the *ggplot2* package (version 3.3.3) [34]. Heatmaps were plotted using the *ComplexHeatmap* package (version 2.6.2) [35]. Venn diagrams were plotted using the *ggVennDiagram* package (version 1.2.0) [36].

#### Expression of intestinal tight junction proteins related to intestinal permeability

RNA was extracted from small intestine tissue using the RNeasy Mini Kit (Qiagen) according to the manufacturer’s protocols. Quality and concentration of RNA was measured using the QIAxpert (Qiagen). Two micrograms of purified RNA were reverse transcribed to complementary DNA (cDNA) using qScript^TM^ XLT cDNA SuperMix (Quantbio) and used for downstream quantitative real-time polymerase chain reaction (qRT-PCR). All primers were obtained from IDT. Gene expression for tight junction protein-1 (TJP1) and occluding were analyzed according to previous real-time PCR work.^11, 12^

The primers were designed using Beacon Designer 3 software.

TJP1: Sense Primer CCATGCCTCCTCCTCCTC,
Anti-sense Primer ACGGAATTGCCTTCACTCTG;
Occludin: Sense Primer GAGGACTGGCTCAGGGAATATC,
Anti-sense Primer TTGTTGACCTCGTCGAGTTCTG.

The two housekeeping genes Ywhaz and Cyca were used for normalization and the 2^-ΔΔCt^ method used as described elsewhere [20]. All samples were run in duplicate on a 384-well plate. Each well contained 20ng of cDNA, 1 X SYBR Green FastMix ROX, and 0.5mM of forward and reverse primers and was run on the QuantStudio system (Qiagen).

## Results

### Validation of microbiome depletion and injury induction

Analyses of bacterial DNA concentration was used to determine if the antibiotic administration was effective at depleting the microbiome. The independent samples t-test demonstrated that animals who consumed the antibiotic cocktail exhibited significant and complete depletion of bacterial DNA from their fecal samples, *t* = 16.678, *p* < .001 Fig 2.

As expected, at the time of the 3^rd^ injury, adult rats weighed more than adolescent rats (F_1, 163_ = 302.89, *p* < .01). There were no weight differences between antibiotic treated and placebo rats (F_1, 163_ = 1.62, *p* = .20) or between rats that experienced RmTBIs and those who received sham injuries (F_1, 163_ = 0.30, *p* = .59), and no significant interactions (*p*’s > .05). See Fig 2.

Following injury induction, the average time-to-right (loss of consciousness) was significantly greater in the RmTBI group when compared to the sham injured group (F_1, 168_ = 175.05 *p* < .01), as well as in the antibiotic treated group as compared to the placebo treated group (F_1, 168_ = 13.30, *p* < .01). Age at the time of injury did not affect average time-to-right, (F_1, 168_ = 0.32, *p* = .57). See Fig 2. Finally, average time spent on the rotarod, which was used to confirm injury induction, was reduced in RmTBI animals compared to sham animals (F_1, 164_ = 14.68, *p* < .01), while also being reduced in adolescents when compared to adults (F_1, 164_ = 39.16, *p* < .01). Antibiotic depletion of the microbiome did not influence time spent on the rotarod, and there were no significant interactions (*p*’s > .05). See Fig 2.

### 16S rRNA gene amplicon sequencing and metabolomics

#### Distinct patterns of microbial and metabolic maturation were identified in healthy adolescents with no maturational differences (stability) identified in adults

**Fig 3:**
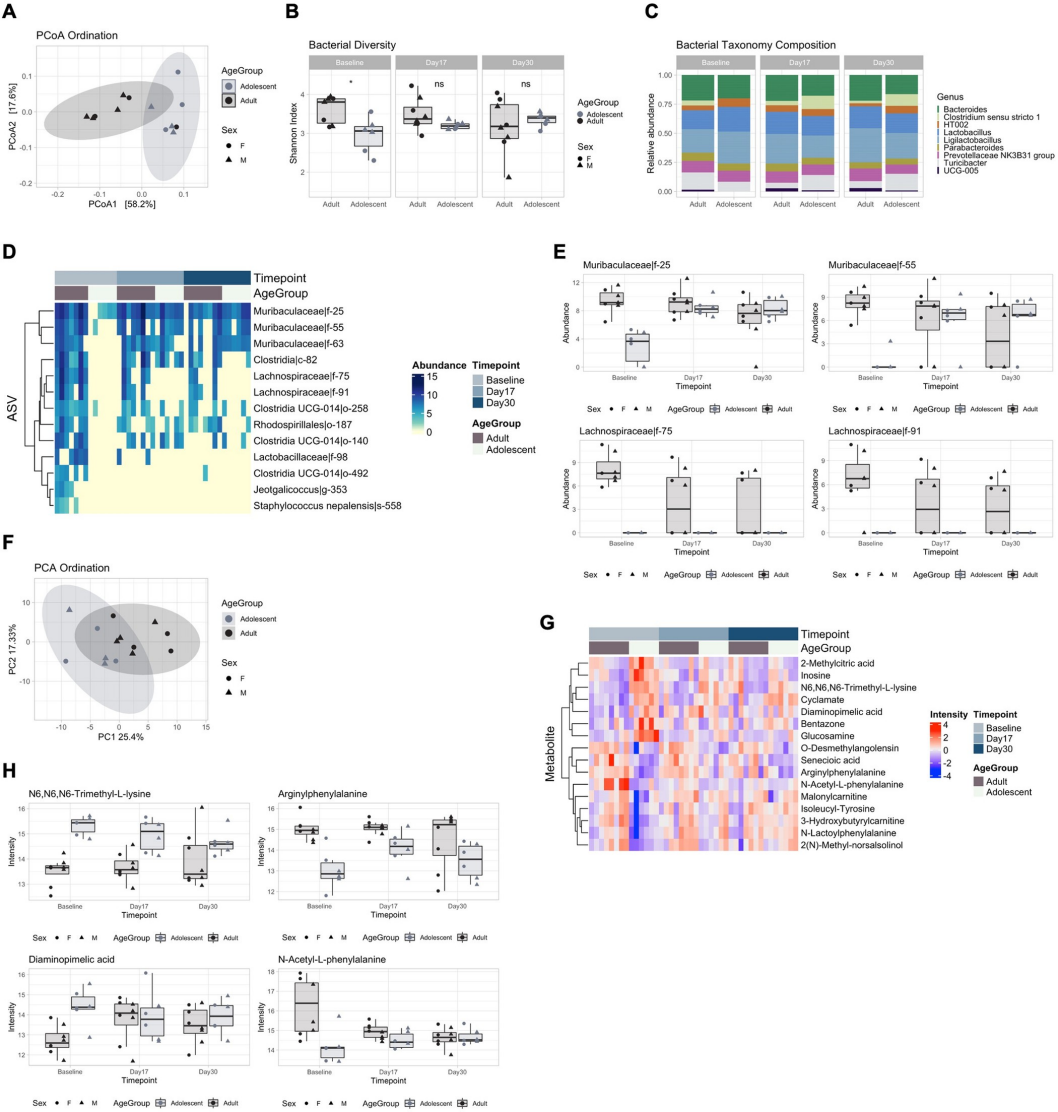
Comparison of healthy adolescent and adult microbial and metabolomic profiles. **(A)** Principal coordinate analysis (PCoA) showing bacterial ordination by age group at baseline. **(B)** Bacterial Shannon diversity boxplots, and **(C)** Taxonomy composition barplots, comparing age groups at baseline, day 17 and day 30. **(D)** Heatmap, and **(E)** Boxplots, of differentially abundant ASVs between age groups over time points. **(F)** Principal component analysis (PCA) showing metabolomic ordination by age group at baseline. **(G)** Heatmap, and **(H)** Boxplots, of differentially abundant metabolites between age groups over time points. Boxplots are indicative of median, interquartile range (IQR) (boxes) and 1.5x IQR (whiskers). *, *p* < 0.05; n.s., not significant.

We first aimed to determine profile differences between adolescents and adults during homeostatic conditions. Bacterial PCoA ordination identified a significant separation between age groups at baseline (Fig 3A, PERMANOVA, R2 = 36.4%, adj. p = 0.002). Likewise, bacterial Shannon diversity was significantly lower (Fig 3B, t-test, p = 0.019) in the adolescent group at baseline compared with the adult group. This increased across the time points and adolescents became similar to the adults from day 17 (t-test, p > 0.094). Bacterial taxonomy was largely composed of the *Bacteroides*, *Clostridium*, *Lactobacillus* and *Ligilactobacillus* genera. The *Clostridium* and *UCG* genera were not detected in adolescents at baseline (Fig 3C). Multivariate analysis using Limma identified 13 ASVs associated with groups and with changes in profile between groups over the time points (Fig 3D). In accordance with Shannon diversity, the largest differences were detected at baseline. Adolescents showed decreased abundance of ASVs within the *Muribaculaceae* family, such as *Muribaculaceae|f-25* (Fig 3E, LFC = -6.51, adj. p = 0.003), highlighting differences in microbial abundance that then normalised by day 17, while *Lachnospiraceae* ASVs such as *Lachnospiraceae|f-75* remained lacking in adolescents (LFC = 8.12, adj. p = 0.003). These data indicate different trends in microbial maturation in adolescents, with “early” and “late” colonisers such as *Muribaculaceae* and *Lachnospiraceae*. Metabolomics trends were in accordance with the microbial changes, with major differences at baseline prior to progression to adult levels (Fig 3F, R2 = 21.3%, adj. p = 0.001) and 16 differentially abundant metabolites (Fig 3G). These included N6,N6,N6 Trimethyl-L-Lysine (Fig 3H, LFC = 1.81, adj. p < 0.001), Arginylphenylalanine (LFC = -1.96, adj. p = 0.002), Diaminopimelic acid (LFC = 1.69 adj. p = 0.0172) and N-Acetyl-L-phenylalanine (LFC = -2.09, adj. p < 0.001). No significant changes in ASVs and metabolites were detected for adults over time or between adolescents and adults at day 17 and day 30. These results suggested a distinct microbial and metabolite profile at baseline, with adults remaining stable over time while adolescents mature to adult levels.

#### Antibiotic treatment may shift the microbiome and metabolome towards a potentially pathogenic proinflammatory profile

**Fig 4:**
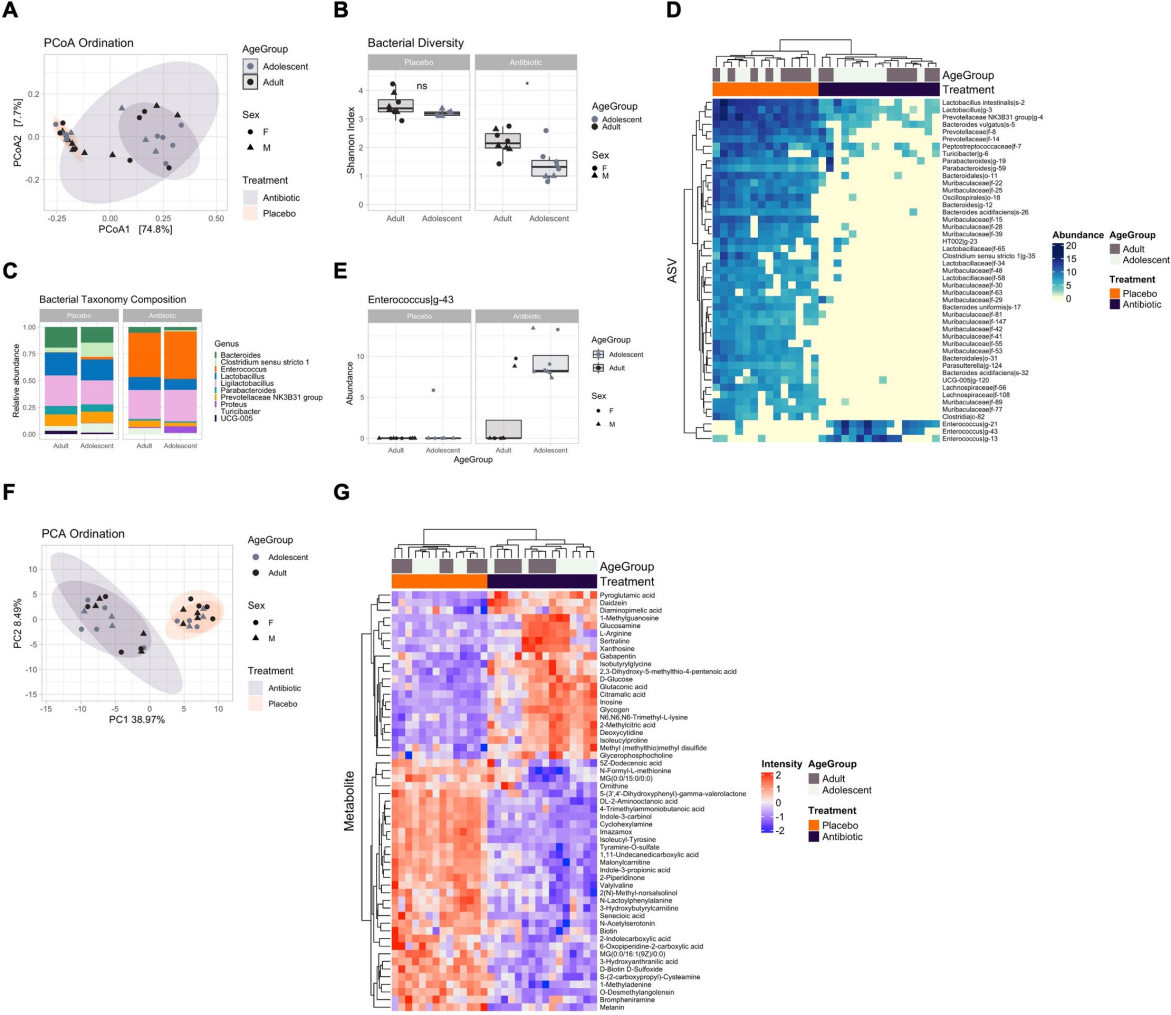
Short-term impact of antibiotics on adolescent and adult microbial and metabolomic profiles. **(A)** Principal coordinate analysis (PCoA) showing bacterial ordination by age group and treatment at day 17. **(B)** Bacterial Shannon diversity boxplots, and **(C)** Taxonomy composition barplots, comparing treatment and age groups at day 17. **(D)** Heatmap of differentially abundant ASVs between treatment groups at day 17. **(E)** Boxplot, of differentially abundant ASVs between treatment and age groups at day 17. **(F)** Principal component analysis (PCA) showing metabolomic ordination by age group and treatment at day 17. **(G)** Heatmap of differentially abundant metabolites between treatment groups at day 17. Boxplots are indicative of median, interquartile range (IQR) (boxes) and 1.5x IQR (whiskers). *, *p* < 0.05; n.s., not significant.

We next sought to determine the short-term impact of antibiotic treatment on adolescents and adults. PCoA ordination significantly separated microbial samples by treatment groups (Fig 4A, PERMANOVA R2 = 54.2%, adj. *p* = 0.001), but not by age groups (R2 = 1.8%, adj. p = 0.229), suggesting a strong effect of antibiotics but no major differences between adolescents and adults. As expected, Shannon diversity was significantly lower in the treatment group (Fig 4B, pairwise t-test, *p* < 0.05 except for Adults Placebo vs Adolescents Placebo), exemplifying the diminished bacterial diversity following antibiotic administration, with a stronger reduction in adolescents compared with adults. Multivariate analysis using Limma identified 47 ASVs associated with the antibiotic treatment at day 17. This resulted in the decrease of *Bacteroides* and *Bifidobacterium* and the increase of *Enterococcus* species (Fig 4C and 4D), with a more pronounced effect in adolescents, which had a significantly higher abundance of *Enterococcus* (LFC = 7.15, adj.p = 0.037) when compared with adults (Fig 4E). PCA ordination of metabolomics samples showed the same separation of samples according to treatment group (Fig 4F, R2 = 46.9%, adj. *p* = 0.001) but not by age groups (R2 = 2.9%, adj. *p* = 0.174). Antibiotic treatment resulted in an altered metabolome profile with 55 differentially abundant metabolites (Fig 4G), and the increase of Xanthosine (LFC = 1.7, adj. *p* = 1.15E-05), Inosine (LFC = 4.67, adj. *p* = 3.30E-09), and L-Arginine (LFC = 2.28, adj. *p* < 0.001), and the decline of Biotin (LFC = -1.22, adj. *p* < 0.001), both also involved in bacterial ABC transporter pathways. No significant differences were detected between adolescents and adults in the antibiotic group, suggesting a similar metabolomic response to the treatment. Overall, this data showed that antibiotic treatment is also accompanied by a profoundly altered metabolic profile involving the increased abundance of metabolites involved in potentially proinflammatory and ABC transporter pathways, a feature of antibiotic-resistant bacteria such as *Enterococcus* [37].

#### The long-term impact of microbiome depletion was stronger for adolescents when compared to adults

**Fig 5:**
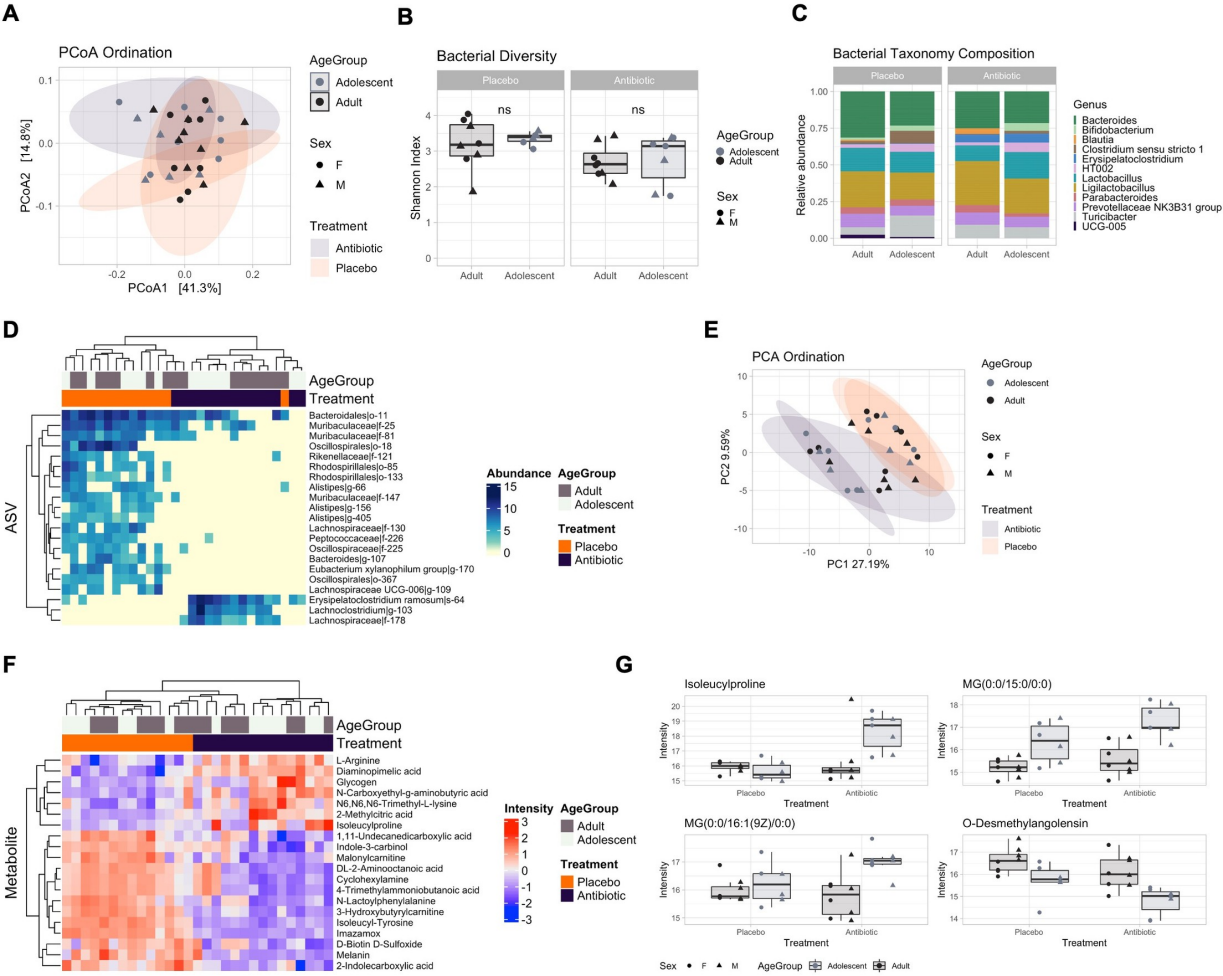
Comparison of antibiotic-treated adolescent and adult microbial and metabolomic profiles. **(A)** Principal coordinate analysis (PCoA) showing bacterial ordination by age group and treatment at day 30. **(B)** Bacterial Shannon diversity boxplots, and **(C)** Taxonomy composition barplots, comparing treatment and age groups at day 30. **(D)** Heatmap of differentially abundant ASVs between treatment groups at day 30. **(E)** Principal component analysis (PCA) showing metabolomic ordination by age group and treatment at day 30. **(F)** Heatmap of differentially abundant metabolites between treatment groups at day 30. **(G)** Boxplots of differentially abundant metabolites between age groups at day 30. Boxplots are indicative of median, interquartile range (IQR) (boxes) and 1.5x IQR (whiskers). n.s., not significant.

We next sought to detect any long-term effects of the antibiotic treatment and whether these were more pronounced in adolescents comparing samples taken at day 30. PCoA ordination showed that after antibiotic treatment microbial profiles of the antibiotic group had largely but incompletely returned to placebo levels (Fig 5A, PERMANOVA R2 = 10.8%, adj. *p* = 0.012) with no differences between age groups (R2 = 5.4%, adj. *p* = 0.11). There was a significant difference in Shannon diversity between treatment groups (Fig 5B, t-test, *p* = 0.0168), suggesting that microbial composition had not completely been restored, but not between age groups (t-test, *p* > 0.5491). An overview of taxonomy composition showed an increase in *Bacteroides* and the disappearance of *Enterococcus* species between treatment groups (Fig 5C). Multivariate analysis using Limma identified 21 ASVs associated with the antibiotic treatment at day 30, of which species such as *Bacteroides*, *Rhodospirillales*, and *Muribaculaceae*, *characteristic of a healthy steady-state*, were found to be significantly decreased, suggesting that at day 30 the microbiome still had not completely returned to placebo levels (Fig 5D). This also included the increase of *Erysipelatochlostridium ramosum*, *Lachnoclostridium*, and *Lachnospiraceae* species, which were not present in the placebo group. However, no differentially abundant ASVs were detected between age groups within the antibiotic-treated group, suggesting a similar response to the treatment. This incomplete return to normal levels was also reflected in the metabolome. PCA ordination showed significant separation between treatment (Fig 5E, R2 = 20.7%, adj. *p* = 0.001) and age groups (R2 = 5.97%, adj. *p* = 0.037). Limma identified 20 metabolites associated with the antibiotic treatment, including L-Arginine (Fig 5F, LFC = 1.11, adj. *p* = 0.001) and Glycogen (LFC = 1.089, adj. p = 0.0138). Contrasting adolescents and adults in the antibiotic group highlighted 4 differentially abundant metabolites, including Isoleucylproline (Fig 5G, LFC = 2.043, adj. *p* = 0.0339) and Monoacylglycerols such as MG(0:0/15:0/0:0) (LFC = 1.74, adj. *p* = 0.0034), significantly increased in adolescents rats, and reduced O-Desmethylagiotensin (LFC = -1.2964, adj. *p* = 0.0325), a bacterial-derived metabolite. These results suggested long-term effects of antibiotic treatment with stronger alterations in the metabolome profile of adolescents.

#### There were transient increases of Faecalibaculum and Lachnospiraceae species following RmTBI in adolescents with potential differences between males and female

**Fig 6:**
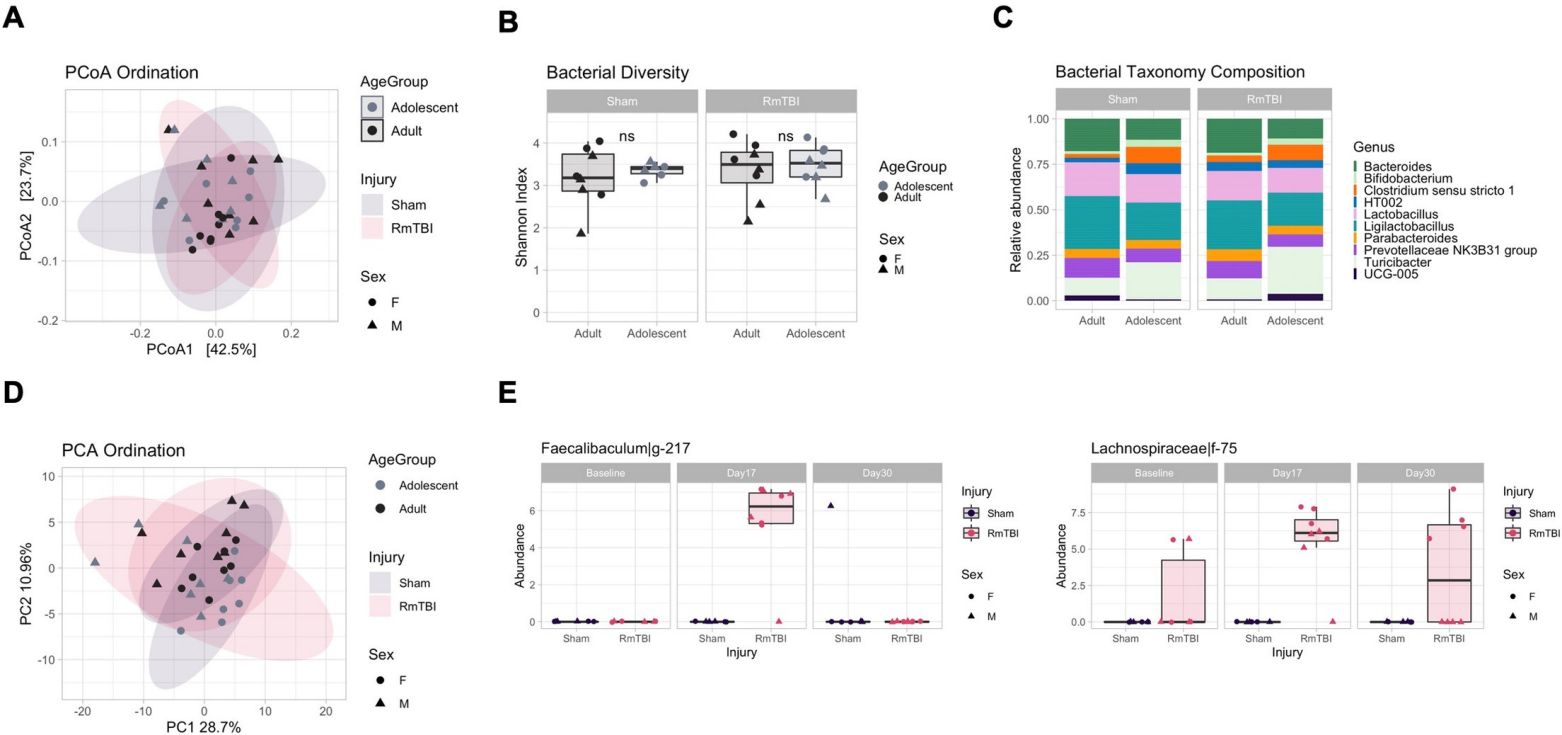
Comparison of placebo RmTBI adolescent and adult microbial and metabolomic profiles. **(A)** Principal coordinate analysis (PCoA) showing bacterial ordination by age group and injury at day 30. **(B)** Bacterial Shannon diversity boxplots, and **(C)** Taxonomy composition barplots, comparing injury and age groups at day 30. **(D)** Principal component analysis (PCA) showing metabolomic ordination by age group and injury at day 30. **(E)** Boxplots of differentially abundant ASVs between adolescent injury groups at day 17. Boxplots are indicative of median, interquartile range (IQR) (boxes) and 1.5x IQR (whiskers). n.s., not significant.

Next, we aimed to characterise the impact of a RmTBI on the placebo group and whether the RmTBI resulted in differential long-term effects between adolescents and adults at day 30. For the microbiome, comparing sham and RmTBI groups showed considerable overlap, with non-significant PCoA ordination (Fig 6A, PERMANOVA, R2 = 1.6%, adj. *p* = 0.852), Shannon diversity (Fig 6B, ANOVA, *p* = 0.434) and taxonomy composition (Fig 6C), which were largely unaffected. No differential ASVs were detected across injury groups. No differences were detected between age groups. This was recapitulated in the metabolome (Fig 6D, R2 = 3.4%, adj. *p* = 0.39) and likely indicated that by day 30 any effects of a RmTBI had resolved. As a consequence, we set out to investigate the immediate effects of a single RmTBI injury at day 17. We detected a significant increase of *Faecalibaculum* (Fig 6E, LFC = 5.52, adj. *p* = 0.0345) and *Lachnospiraceae* (LFC = 5.68, adj. *p* = 0.0345) in adolescents RmTBI, suggesting a rapid short-term effect at day 17. The *Lachnospiraceae* species remained increased in females at day 30, while it diminished in males, suggesting differential long-term responses to RmTBI in males and females.

#### The RmTBI did not influence microbiome composition or function in the antibiotic groups

**Fig 7:**
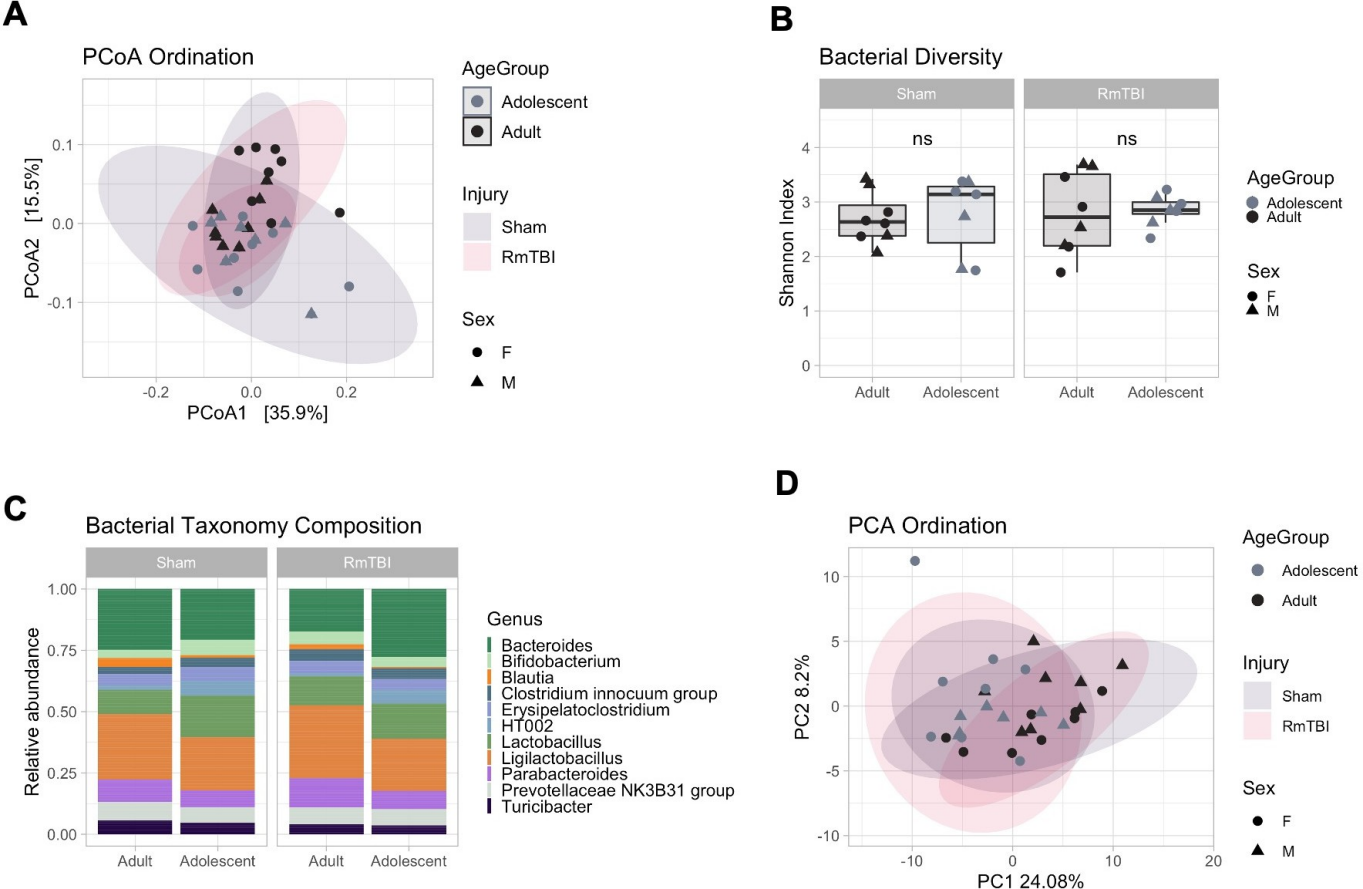
Comparison of antibiotic RmTBI adolescent and adult microbial and metabolomic profiles. **(A)** Principal coordinate analysis (PCoA) showing bacterial ordination by age group and injury at day 30. **(B)** Bacterial Shannon diversity boxplots, and **(C)** Taxonomy composition barplots, comparing injury and age groups at day 30. **(D)** Principal component analysis (PCA) showing metabolomic ordination by age group and injury at day 30. Boxplots are indicative of median, interquartile range (IQR) (boxes) and 1.5x IQR (whiskers). n.s., not significant.

We further aimed to characterise the effect of a RmTBI in the antibiotic group at day 30. Comparison of antibiotic RmTBI with antibiotic Sham showed complete overlap between injury groups (Fig 7A, PERMANOVA, R2 = 1.7%, adj. *p* = 0.817), and no changes in bacterial Shannon diversity (Fig 7B, ANOVA, *p* = 0.669) and composition (Fig 7C). No differential ASVs were detected between groups. A similar outcome was seen for the metabolome profile (Fig 7D, R2 = 2.1%, adj. p = 0.844), suggesting that the RmTBI did not affect the metabolome either. We postulate that any microbial alterations following RmTBI could have been missed due to the diminished abundance of bacterial species caused by antibiotic treatment. Changes to the metabolome could have been transient and had likely resolved by day 30.

#### The effects of antibiotics were exacerbated by a RmTBI

**Fig 8:**
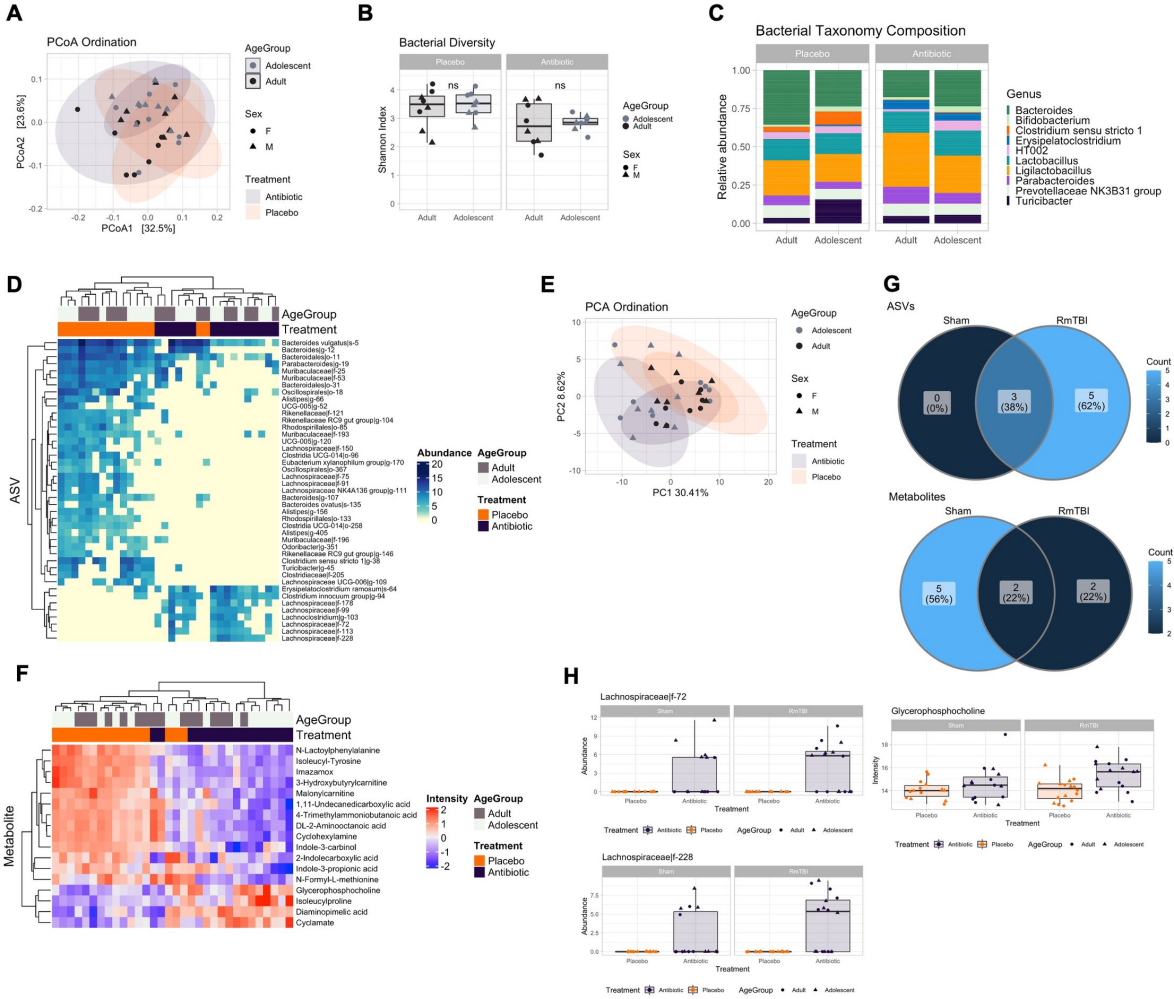
Comparison of antibiotic and placebo RmTBI adolescent and adult microbial and metabolomic profiles. **(A)** Principal coordinate analysis (PCoA) showing bacterial ordination by age group and treatment at day 30. **(B)** Bacterial Shannon diversity boxplots, and **(C)** Taxonomy composition barplots, comparing treatment and age groups at day 30. **(D)** Heatmap of differentially abundant ASVs between treatment groups at day 30. **(E)** Principal component analysis (PCA) showing metabolomic ordination by age group and treatment at day 30. **(F)** Heatmap of differentially abundant metabolites between treatment groups at day 30. **(G)** Venn diagrams showing unique hits to the antibiotic groups. Top: ASVs. Bottom: Metabolites. **(H)** Boxplots of differentially abundant ASVs (left) and metabolites (right) unique to the antibiotic RmTBI group at day 30, compared with trends in the Sham group. Boxplots are indicative of median, interquartile range (IQR) (boxes) and 1.5x IQR (whiskers). n.s., not significant.

We next sought to determine how undergoing a RmTBI following microbiome depletion could have affected microbial and metabolite profiles. For this purpose, we first compared antibiotic RmTBI vs placebo RmTBI at day 30. Similar to the Sham group (Fig 5), PCoA ordination for the RmTBI group showed a significant separation between treatments (Fig 8A, PERMANOVA, R2 = 12%, adj. *p* = 0.001). In contrast to the Sham group (Fig 5), we also detected a significant separation between age groups (R2 = 6.4%, adj. *p* = 0.026), but no interaction between injury and age (R2 = 3.1%, adj. *p* = 0.31). Shannon diversity between placebo and antibiotic groups was also significant (Fig 8B, t-test, *p* = 0.005), but not between age groups (t-test, *p* > 0.64). We found 43 differentially abundant ASVs, with increased *Erysipelatoclostridium* and *Lachnospiraceae* species between treatment groups (Fig 8C and 8D). PCA ordination of the metabolome dataset showed significant separation between treatment (Fig 8E, R2 = 16%, adj. *p* = 0.001), age groups (R2 = 7.7%, adj. *p* = 0.011), but no interactive effect of both (R2 = 3.2%, adj. *p* = 0.239). We identified 17 differentially abundant metabolites between antibiotic and placebo RmTBI groups (Fig 8F). Species and metabolites upregulated in the antibiotics group largely overlapped with those significant in the Sham group comparison (Fig 5D–5F), with the exception of 5 ASVs and 2 metabolites that were uniquely upregulated in the RmTBI group (Fig 8G), while not reaching significance in the Sham group (Fig 8H). Interestingly, four of the 5 unique ASVs were *Lachnospiraceae* species such as *Lachnospiraceae|f-72* (LFC = 4, adj. p = 0.0187), while one of the unique metabolites, Glycerophosphocholine (LFC = 1.3114, adj. *p* = 0.0123), was found to be increased in the RmTBI group. These results suggested that RmTBI following antibiotic treatment could have resulted in an enhanced long-term antibiotic effect, with the enhanced abundance of *Lachnospiraceae* species.

### Intestinal tight junction protein expression

**Fig 9:**
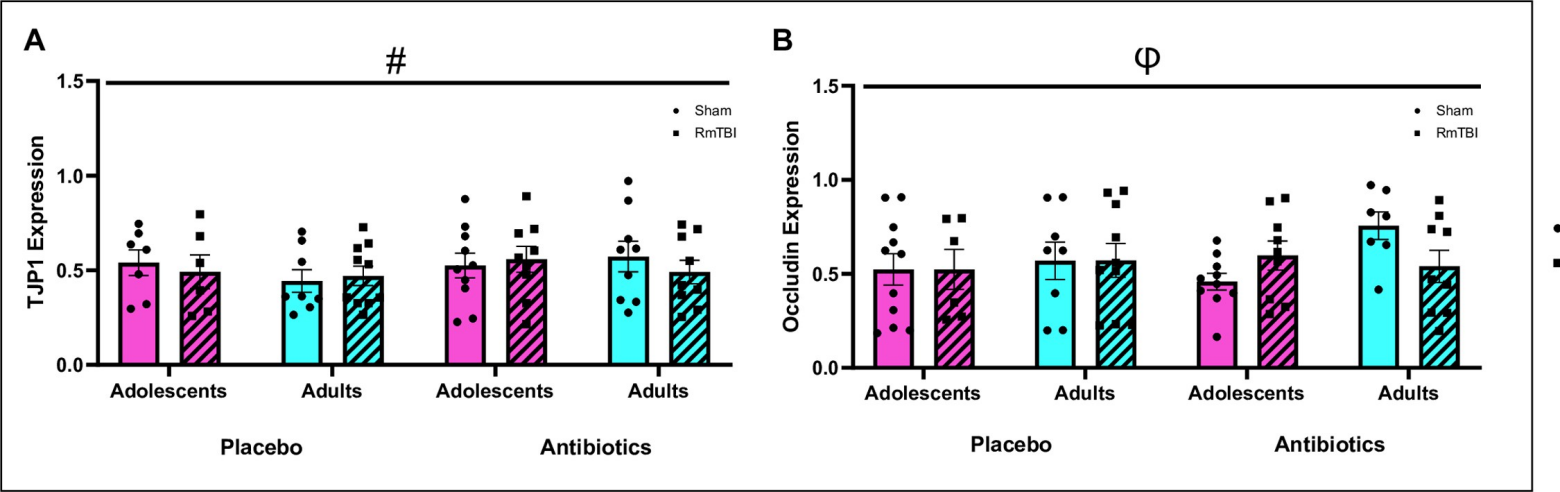
Gene expression of intestinal tight junction proteins TJP1 and Occludin. Means and individual data points ± standard error are shown. (#) indicates a main effect of treatment, (φ) indicates a significant interaction; *p*’s < .05. A) Displays *TJP1* concentration, whereby antibiotics groups had increased expression of TJP1, B) Displays *occludin* concentration, whereby interactions observed were Injury*Age whereby expression was increased in placebo+RmTBI and antibiotic+RmTBI adolescent rats and reduced in placebo+RmTBI and antibiotic+RmTBI adult rats.

At day 30 (11 days post last RmTBI and 16 days post antibiotic cocktail cessation) intestinal tight junction protein expression was analysed. We identified increased *TJP1* expression in microbiome depleted adolescent and adult rats (*p <* .*05)*, but no change in *TJP1* expression in RmTBI adolescent or adult rats and there were no differences between adolescents and adult rats (*p’s >* .*05)*. Conversely, we identified an injury by age interaction for *occludin* expression. *Occludin* expression was increased in adolescent RmTBI+Placebo and RmTBI+Antibiotic adolescent rats but was reduced in adult RmTBI+Placebo and RmTBI+Antibiotic rats, *p’s <* .*05*. See Fig 9.

## Discussion

Previous studies have demonstrated that TBI alters gut microbiome composition [17,18], function [12], and intestinal permeability [38]. However, most preclinical [4,17,38–41] and clinical studies [42] investigate the impact of TBI on the adult gut microbiome ignoring the adolescent time period, as well as the possible implications of gut microbiome dysfunction prior to TBI. Therefore, the present study sought to investigate the comparative effects of microbiome depletion prior to RmTBI on microbiome composition and functionality in adolescents and adult rats. We also explored the possibility that microbiome depletion prior to injury would exacerbate changes to intestinal permeability. First, our findings demonstrate a microbial maturational change in adolescents that reach adult levels by P51, but a stability over the experimental time frame for adult rats. Given that depletion of the gut microbiome is a common modality utilised to investigate the role of the microbiome [43], we used 14-day antibiotic administration prior to injury, as a mechanism to determine if the microbiome is involved in mTBI pathophysiology, and how microbiome diversity and functionality recovers in the context of RmTBI. Second, we demonstrate that microbiome depletion significantly modified the adolescent microbiome and metabolome, both at the acute and chronic timepoints of our examination. Importantly, the changes identified following microbiome depletion were more pronounced in adolescents than adults. Third, and contrary to our hypothesis, RmTBI only modified the microbiome and metabolome transiently, and these changes were only identified in adolescents. Finally, microbiome depletion prior to RmTBI induced chronic changes to the microbiome composition and metabolome in both adolescent and adult rats. To our knowledge, this is the first study to demonstrate that following microbiome depletion, differential repopulation of gut bacterial species and their functioning metabolites occurred post-RmTBI in adolescent and adult rats, with adolescents exhibiting greater changes to microbiome diversity, composition, and metabolomic changes. Furthermore, as a marker of intestinal permeability, expression levels of tight junction proteins were altered in response to microbiome depletion (*TJP1*) and differentially altered between adolescents and adults following RmTBI (*Occludin*).

### Microbial and metabolic maturation in healthy adolescents versus stability in adults

Recent literature has demonstrated that throughout childhood and into adulthood, the microbiome undergoes significant development [44,45]. The adolescent period in particular is an important critical window in microbiome maturation [46]. Therefore, in order to control for any potential age specific maturational changes over the experimental window, we completed comparative analysis of microbiome diversity, composition, and metabolomics in adolescents and adults from our control groups. Interestingly, 16S rRNA gene amplicon sequencing demonstrated microbial stability in adult rats with adolescents exhibiting microbial and metabolomic maturation over time. Specifically, we saw significant differences in bacterial species and metabolites at baseline between adolescents (P21) and adults (P100), with adolescents exhibiting reduced abundance of the *Muribaculaceae* family of bacteria, that reached adult levels by P37 and a complete absence of *Lachnospiraceae* from baseline to P51. To the best of our knowledge, this is the first study to compare adolescent and adult microbial composition over time and demonstrate microbial maturation in adolescents. The metabolomic profile was also different at baseline in adolescents and adults, with bacterial metabolites involved in L-cartinine biosynthesis [47] and L-lysine biosynthesis [48] (N6,N6,N6-Trimethyl-L-lysine and Diaminopimelic acid (DAPA), respectively) being increased at baseline in adolescents but displaying adult levels by P37. These bacterial metabolites are associated with the development of the intestinal mucosa and intestinal enterocytes (N6,N6,N6-Trimethyl-L-lysine) via the regulation of short chain fatty acids (SCFAs) [47,49] and the structural organization of gram-negative gut bacterial cell walls (DAPA) [48]. Importantly, DAPA is a vital component of gram-negative bacterial peptidoglycan [48] like those of the *Muribaculaceae* family [50–52]. Considering this, it is possible that these particular metabolites are elevated at the beginning of adolescence to drive microbiome development and diversity to adult levels which may be attributed to change in diet induced by weaning. Recent studies have shown metabolites involved in phenylalanine pathways, that are produced by *Clostridium* and *Lachnospiraceae* bacteria, have been found to be important in intestinal membrane integrity [53,54]. As these bacterial species were reduced in adolescents at baseline, this may be responsible for the initial reduction in phenylalanine derived metabolites. However, to date there is a gap in the literature exploring gut derived metabolic profiles, specifically in adolescents across this developmental period.

### Acute and chronic influence of microbiome depletion on the composition and function of the gut microbiome in adolescents and adults

In preclinical studies, the administration of a broad-spectrum antibiotic cocktail over a 14-day period is commonly utilized as a method of depleting enteric bacteria with low systemic absorption [38,43,55,56]. However, there have been no studies that have investigated this on the adolescent microbiome and its comparative effects to adults. In the present study, both adolescent and adult microbiome composition were significantly modified by the antibiotic cocktail at P37/P116 (4 days post-antibiotic cessation), with a loss of most bacterial species. Importantly, we identified substantial reductions in ‘beneficial’ bacteria including *Bacteroides* and *Bifidobacterium*, while also finding increases in the potentially pathogenic *Enterococcus*; with *Enterococcus* being significantly more abundant in adolescent rats than adults. Consistent with our results, previous preclinical studies have also found increases in *Enterococcus* post-microbiome depletion in adult rodents [57] and post-antibiotic treatment in clinical studies [58]. As *Enterococcus* is associated with a wide range of pro-inflammatory conditions and infections in hospital settings [59,60], it is possible that early life chronic exposure to antibiotics, particularly in adolescence may pose as a predictive factor of pro-inflammatory conditions later in life.

Changes to the metabolome were also seen in both adolescent and adult rats, with increases in metabolites involved in metabolism of purines (inosine and xanthosine) [61]. Purine metabolism metabolites are involved in pro-inflammatory processes [37] further suggesting a potentially pro-inflammatory state acutely following the after microbiome depletion, which may have resulted in the altered metabolomic profile. Although these effects were seen transiently, it appears that adolescents were affected to a greater extent by the antibiotic administration, with their bacterial diversity remaining lower at P51 (16 days post-microbiome depletion). Specifically, reductions in *Bacteroides*, *Rhodospirillales*, and *Muribaculaceae*, which are common in the healthy steady state, were significantly decreased at P51 in adolescents and the potentially opportunistic species *Erysipelatoclostridium ramosum*, *Lachnoclostridium*, and *Lachnospiraceae* were increased. Although the role of *Lachnospiraceae* in health and disease is controversial [62], as it was not present in our adolescent controls, it may be a compensatory response to restore balance, as it is involved in anti-inflammatory processes via the production of SCFAs like butyrate [63]. However, this overabundance of the family of ‘beneficial’ bacteria, has also been associated with an aging microbiome [64] and found to be in increased abundance in individuals with metabolic disorders [65]. Therefore, as this increase in *Lachnospiraceae* was not identified across the adolescent microbial maturation, it may be a compensatory or detrimental consequence in an attempt to restore balance to the previously depleted microbiome.

Research suggests that the initial state of an individual’s microbiome composition may shape their microbiome profile after antibiotic administration [66]. Specifically, previous clinical studies have demonstrated that those with lower bacterial diversity prior to antibiotic administration may be more susceptible to opportunistic/pathogenic species such as *Lachnoclostridium* [66]. Given that adolescents had less diversity at baseline (prior to antibiotic administration), this may have contributed to the increased *Erysipelatoclostridium ramosum* and *Lachnoclostridium* we identified following cessation of the antibiotics. We also identified a higher abundance of isoleucylproline and monoacylglycerols in microbiome depleted adolescent rats. Interestingly, monoacylglycerols have been associated with anti-microbial properties [67,68] and may be a compensatory action to reduce the abundance of opportunistic bacteria. A reduction in O-desmethylangolensin (O-DMA), a bacterial metabolite involved in the breakdown of certain soy containing foods [69], was reduced in adolescents compared to adult rats at day 30. In order to produce O-DMA in response to dietary consumption of soy isoflavones, appropriate fermentation by intestinal bacteria is required [70]. The appropriate fermentation of these isoflavones (phytoestrogens) are important for the regulation of inflammatory signal pathways, intestinal barrier function, and general microbiome composition balance [71,72]. Therefore, although the bacterial composition appeared to be relatively restored, the functionality of bacteria that were present may not be optimal in adolescents. This is further exemplified in our analyses of tight junction proteins, where *TJP1* exhibited increased expression in both adolescent and adult microbiome depleted rats. Previous studies have found that 14-day antibiotic administration reduces *TJP1* expression, however, most have investigated tight junction expression at an acute timepoint, immediately after administration is ceased [38,73–75]. Considering we measured *TJP1* expression at a more chronic time point (16 days after ceasing antibiotic administration) it is possible that the increase may be a compensatory mechanism or a delayed function of gut bacteria recolonisation to rebuild the gut lining after dysbiosis. Intriguingly, we did not observe any changes to the expression of occludin 16 days post antibiotic administration. Previous studies have also shown that microbiome depletion does not cause changes to *occludin* expression [76]. However, there is a plethora of literature that have investigated the impact of TBI on intestinal membrane permeability/integrity which are prominently modelled in adult rodents at a chronic timepoint. As in the present study, reduced intestinal *occludin* expression after injury in adult rodents may be attributed to the induction of pro-inflammatory cytokines such as TNF-α to intestinal epithelial cell receptors resulting in mucosal injury [3,77]. Unexpectedly, an increase in *occludin* expression was found in adolescent RmTBI rats, which may be attributed to the increase in ‘beneficial butyrate producing bacteria’ in order to maintain gut barrier integrity [63,78–80].

### There is a transient increase of Faecalibaculum and Lachnospiraceae species following RmTBI in adolescents

Rodent models of RmTBI have demonstrated microbiome dysbiosis in a time-dependent manner, with significant changes being observed as early as 2 hours after mTBI [41]. The present study sought to investigate how microbiome depletion prior to RmTBI could influence adolescent and adult rats at two time points (day 17 and day 30). At day 17, placebo treated adolescent rats exposed to RmTBI, exhibited transient increases in abundance of *Faecalibaculum* and *Lachnospiraceae*. Importantly, no changes to the metabolome were seen in adolescent or adult rats both acutely or chronically post-RmTBI. In a similar study, Angoa-Perez et al, [17] exposed mice to RmTBI and found transient and minimal change to the microbiome. The authors attributed this to the mild nature of the injuries which may not have produced enough systemic damage to see robust changes to microbiome composition. Increases in bacteria belonging to the Erysipelotrichaceae family such as *Faecalibaculum*, as well as increases in *Lachnospiraceae*, both of which are considered ‘beneficial butyrate producers’ have been shown to be increased following mTBI in rodents [40] as seen in the present study. Conversely, it is commonly reported that following severe TBI there is a reduction in these ‘beneficial’ bacteria, up to 30 days following injury [18,81]. The exact mechanisms driving this increase in beneficial butyrate producing bacteria after injury is unknown, however, it may be a protective response to compensate for the secondary injury cascade that follows mTBI [82,83]. It is important to note here that the role of *Lachnospiraceae* in health and disease is controversial [62], with overabundance being associated with dysbiosis, neurodegenerative disease [84], and metabolic disorders [85].

Although not the focus of these experiments, we also identified a persistent increase in *Lachnospiraceae* at P51 in female adolescent RmTBI rats. There is currently no literature investigating the influence of sex on microbiome composition after RmTBI. Moreover, there is minimal literature demonstrating sexual dimorphism in *Lachnospiraceae* abundance. Considering females are more likely to experience worse symptomology following mild to severe TBI [86], this persistent increase in *Lachnospiraceae* and therefore persistent dysbiosis, may potentially indicate a greater susceptibility or vulnerability to the effects of RmTBI. However, more studies are required to understand the sexually dimorphic effects of RmTBI on the microbiome.

### Microbiome depletion prior to RmTBI exacerbates microbiome dysbiosis and function

In the present study adolescent and adult rats with their microbiome depleted prior to RmTBI demonstrated significant changes to the microbiome composition and metabolome. At day 30 we identified an increase in *Erysipelatoclostridium* and *clostridium innocuum* and a decrease in *Bacteroides* and *Clostridium sensu stricto*. Changes to microbiome composition were modified to a similar degree in both RmTBI adolescents and adults. Reductions in *Bacteroides* have been associated with irritable bowel syndrome (IBS) development and identified after stroke [87]. *Bacteroides* are imperative for the maintenance of intestinal barrier integrity, with supplementation being associated with increased tight junction proteins [88]. Reductions in *Clostridium sensu stricto* have been associated with reduced butyrate production and Alzheimer’s disease [89,90]. We therefore speculate that depletion of the microbiome prior to RmTBI may further induce pro-inflammatory cascades both systemically and in the gut. Increased *Clostridium innocuum* was also seen in microbiome depleted RmTBI rats, which has been linked to antibiotic induced diarrhoea and colitis [91,92]. *Erysipelatoclostridium* is a potentially opportunistic species and when increased is considered to be a biomarker for Crohn’s and gastrointestinal infections [93,94], while also being associated with schizophrenia [95], Parkinson’s disease [96], and reduced serotonin production [97].

Within the metabolome, we identified reductions in indole-3-carbinol which has been associated with obesity, certain types of cancer, cardiovascular disease, insulin resistance [98], colitis, and microbial dysbiosis [99], further demonstrating a potentially chronic and exacerbated inflammatory response to microbiome depletion prior to injury. Furthermore, we observed increased *glycerophosphocholine* in adolescent microbiome depleted RmTBI rats which has been associated with increased risk of cardiovascular disease development later in life [100]. Therefore, it is possible that the functionality of the microbiome within our adolescents was modified to a greater extent than adults by the RmTBI.

## Conclusion

In our endeavour to understand how microbiome depletion prior to RmTBI altered the microbiome composition and function in adolescents and adult rats, we made multiple unique and important findings. First, we demonstrate that the adolescent microbiome and microbial metabolome undergoes significant maturation and is not comparable to adulthood until P51. Although there have been numerous studies examining the microbiome early in life, this is one of the first to show composition and functional differences between adolescents and adults. Second, antibiotic administration exhibited greater influence on microbiome recovery in adolescents suggesting that antibiotic treatment may have detrimental effects for long-term health when administered during this developmental period. While antibiotic administration is often necessary and vital for many infections, we may need to consider simultaneous prophylactic pre- and pro-biotic therapy. Finally, we corroborated previous studies showing that RmTBI does not result in substantial disruption to the microbiome. Unlike moderate-to-severe TBI that has been associated with significant modification to intestinal permeability, mTBI and RmTBI do not appear to produce “leaky gut” which may prevent microbial dysbiosis. To further elucidate the impact of microbial alterations and RmTBI on the functioning of the intestinal environment and gut barrier, we could have assessed protein levels in addition to qPCR and this should be explored in future studies. In addition, long-term changes in the gut-brain-immune axis associated with RmTBI should be investigated, as the majority of current studies, including ours, have focused on the acute and subacute timeframes.
